# Assessment of Metabolome Annotation Quality: A Method for Evaluating the False Discovery Rate of Elemental Composition Searches

**DOI:** 10.1371/journal.pone.0007490

**Published:** 2009-10-16

**Authors:** Fumio Matsuda, Yoko Shinbo, Akira Oikawa, Masami Yokota Hirai, Oliver Fiehn, Shigehiko Kanaya, Kazuki Saito

**Affiliations:** 1 Metabolome Research Group, RIKEN Plant Science Center, Yokohama, Kanagawa, Japan; 2 Graduate School of Information Science, Nara Institute of Science and Technology, Ikoma, Nara, Japan; 3 Japan Science and Technology Agency, CREST, Kawaguchi, Saitama, Japan; 4 Metabolomics Research Laboratory, UC Davis Genome Center, Davis, California, United States of America; 5 Graduate School of Pharmaceutical Sciences, Chiba University, Chiba, Japan; Cairo University, Egypt

## Abstract

**Background:**

In metabolomics researches using mass spectrometry (MS), systematic searching of high-resolution mass data against compound databases is often the first step of metabolite annotation to determine elemental compositions possessing similar theoretical mass numbers. However, incorrect hits derived from errors in mass analyses will be included in the results of elemental composition searches. To assess the quality of peak annotation information, a novel methodology for false discovery rates (FDR) evaluation is presented in this study. Based on the FDR analyses, several aspects of an elemental composition search, including setting a threshold, estimating FDR, and the types of elemental composition databases most reliable for searching are discussed.

**Methodology/Principal Findings:**

The FDR can be determined from one measured value (i.e., the hit rate for search queries) and four parameters determined by Monte Carlo simulation. The results indicate that relatively high FDR values (30–50%) were obtained when searching time-of-flight (TOF)/MS data using the KNApSAcK and KEGG databases. In addition, searches against large all-in-one databases (e.g., PubChem) always produced unacceptable results (FDR >70%). The estimated FDRs suggest that the quality of search results can be improved not only by performing more accurate mass analysis but also by modifying the properties of the compound database. A theoretical analysis indicates that FDR could be improved by using compound database with smaller but higher completeness entries.

**Conclusions/Significance:**

High accuracy mass analysis, such as Fourier transform (FT)-MS, is needed for reliable annotation (FDR <10%). In addition, a small, customized compound database is preferable for high-quality annotation of metabolome data.

## Introduction

In recent metabolomics studies using mass spectrometry (MS), advances in high-resolution MS, including time-of-flight (TOF)- [1], Orbitrap- [2], and Fourier transform ion cyclotron resonance (FT-ICR)-MS [3], have made it possible to acquire metabolome data with accurate mass-to-charge ratios (*m*/*z*) [4]–[6]. In metabolomics research using metabolic fingerprinting and differential metabolomics techniques, such as disease diagnosis [7] and marker discovery [8], structural elucidation of no or only a small number of selected metabolites may be performed, because the primary goal of the analysis is evaluation of similarities and/or differences in the entire metabolome dataset across samples. On the other hand, comprehensive annotation of metabolite signals is required in metabolomics research to describe a metabolic event occurring in a target organ in as detailed a manner as possible. However, many metabolite signals in raw metabolome data cannot be identified through chromatographic and spectroscopic comparison with that of standards, especially in plant metabolomics studies dealing with secondary metabolites [3], [9]. To elucidate the structure of metabolite signals prior to the isolation of metabolites, MS data, including tandem mass spectra and high-resolution mass data, has been utilized [4]. Whereas the acquisition of MS/MS spectral data often requires additional effort, high-resolution mass data are available from the metabolic profile data itself. Thus, systematic searching of high-resolution mass data against compound databases is often the first step of metabolite annotation to determine elemental compositions possessing similar theoretical mass numbers [3], [10]–[15]. The deduced elemental compositions are then adopted for “identification” or “annotation” of metabolome signals using the compound nomenclature system proposed by the Metabolome Standard Initiative (MSI) [16]. Although putative elemental compositions could be assigned to many metabolite signals using these methods, it should be noted that incorrect hits (i.e., false positives) derived from errors in mass analyses will be included in the search results [17]. When the false positive rate for the elemental composition search results as a whole is relatively high, caution should be used in applying the search results for metabolite annotation. In addition, quality evaluation of the search results is essential in understanding the basic aspects of the elemental composition search, including setting a suitable threshold, the accuracy of MS analysis required for reliable searching, and the types of elemental composition databases that will provide the most reliable results. Therefore, evaluation of false discovery rates (FDRs) in elemental composition search results is essential to minimize misinterpretation of metabolome data.

Despite its importance, the FDR issue has not been sufficiently considered, likely due to lack of relevant methodology. In the field of proteomics, FDRs have been estimated for peptide identification results derived from database searches of peptide MS/MS spectra [18]. A commonly used method is to search the set of peptide MS/MS spectra against an original (target) protein database as well as a decoy database, which is a database of reversed amino acid sequences of the target database [19], [20]. Because hits in decoy databases are random, FDRs have been determined by comparing the number of query hits in the decoy and target databases [21]. The decoy data must be conceivable peptides, but never an exact result of a search; therefore, a complete peptide database created from the genome sequence is needed. On the other hand, the creation of a decoy database for an elemental composition library would likely be difficult, because a “decoy” molecular formula (a compound-like formula) could not be distinguished from the formula of an actual metabolite. Thus, a different strategy is required to deduce the FDR for the results of an elemental composition search. In this study, a novel methodology for FDR evaluation is presented, considering several aspects of an elemental composition search against a compound database.

## Results

### Density and completeness of compound databases

**Figure 1:**
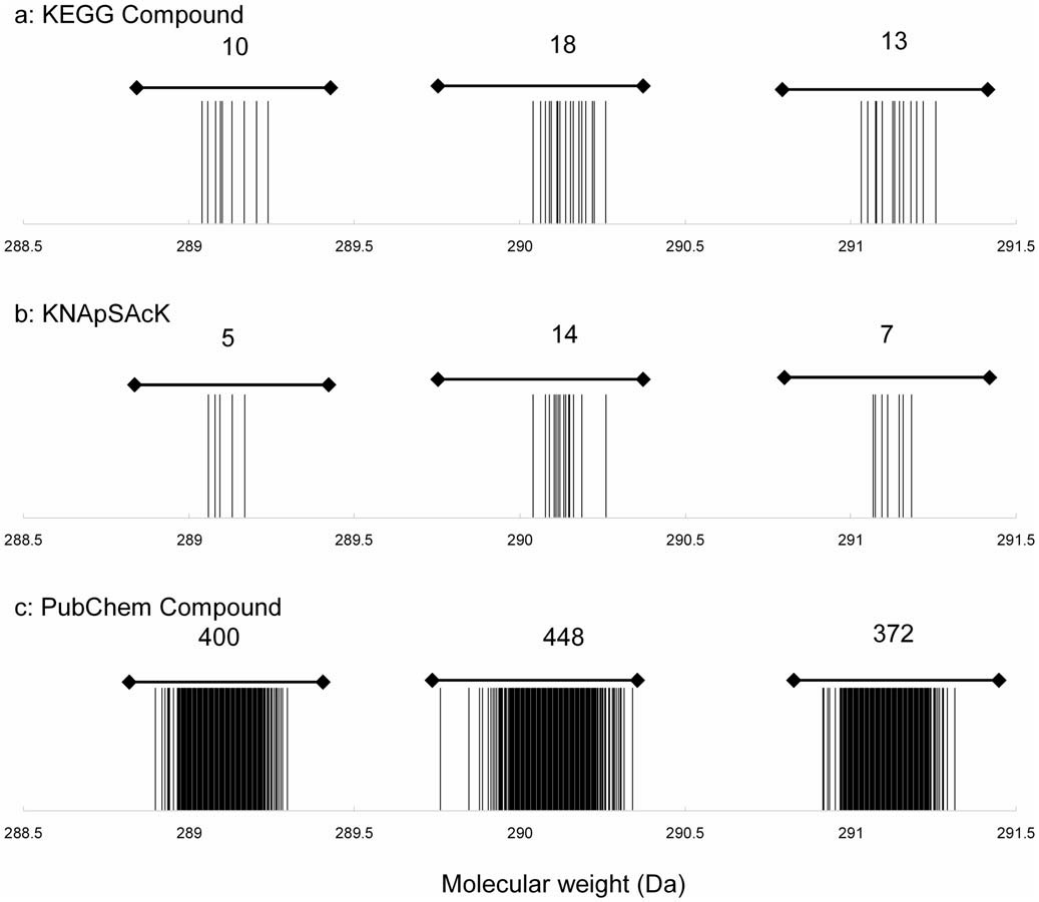
Density of unique molecular formula (weight) data around 289, 290, and 291 Da. (A) KEGG Compound (12,382 compounds and 5,547 unique molecular formulae comprising C, H, N, O, S, and P), (B) KNApSAcK (23,127 compounds and 6,544 unique formulae), and (C) PubChem Compound (19,140,080 compounds and 473,108 unique molecular formulae) datasets. Vertical lines represent the monoisotopic molecular weights for each entry. The total number of molecular formulae near each molecular weight is indicated above the group of vertical lines.

FDRs of elemental composition search results are expected to be affected by three factors: (i) accuracy of the mass analysis of the query metabolome data (σ), (ii) width of the threshold for searching (Δ_thres_), and (iii) the properties of the compound database. When the analytical error is larger than the threshold value, the molecular formula search will not obtain a correct answer from the database (i.e., a false negative). The false negative rate can be estimated from (i) the mass accuracy and (ii) the search threshold. For example, when the Δ_thres_ = 2.0σ, false negatives can be deduced to be approximately 5%, assuming a normal distribution of mass analysis errors. On the other hand, the frequency of false positives depends on the “density” of the database (the number of molecular formulae within a specific nominal mass window), in addition to the mass accuracy and search threshold. As shown in Figure 1, in contrast to two databases with sparse molecular formula entries, KEGG Compound [22] and KNApSAcK [23] (5,547 and 6,544 unique molecular formulae, respectively), PubChem (473,108 formulae) [24] is very crowded with an abundance of molecular-formula entries. This density suggests that a PubChem search will produce a larger number of false positives than searching against either KEGG or KNApSAcK (Fig. 1).

**Figure 2:**
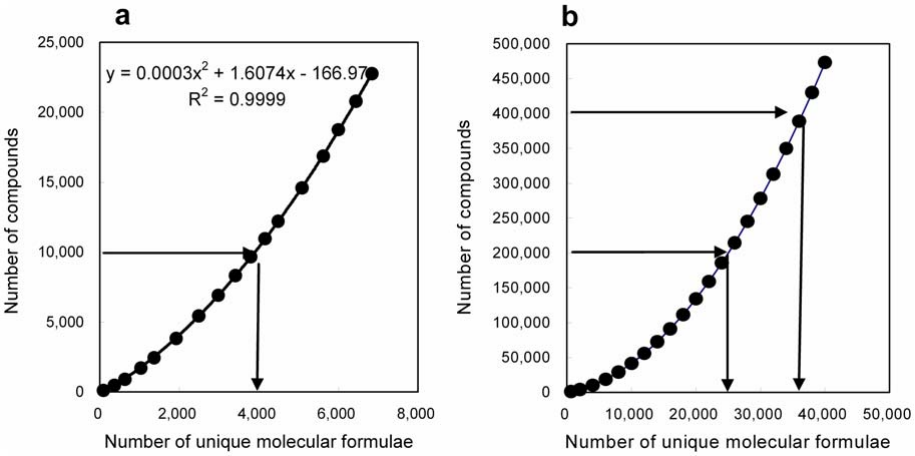
Simulated growth curve for KNApSAcK data. (A) Simulated relationship between the number of compounds in KNApSAcK (y-axis) and unique molecular formulae (x-axis). For example, 10,000 compounds were arbitrarily extracted from the pool of all KNApSAcK entries (23,127 compounds) without repetition, and the number of unique molecular formulae was counted. This procedure was repeated 1,000 times to obtain the average number of unique molecular formulae (3,912) associated with 10,000 compounds. Following the determination of expected numbers of formulae for various numbers of compounds, a simulated growth curve for KNApSAcK data was generated. The equation for the approximated curve (*R* ^2^ = 9.9999) is also shown. (B) The predicted growth curve for future KNApSAcK versions. The numbers of unique molecular formulae in future KNApSAcK databases were estimated through extrapolation of the simulated curve.

In addition, the “completeness” of the compound database must be taken into consideration, because relatively small compound databases such as KEGG and KNApSAcK may not provide exact results, due to having incomplete collections of compound data. Even if a compound database does not include an exact match, false positives can occur. In this study, the completeness of compound databases for plant metabolomics studies was roughly estimated using the following procedure. The current version of KNApSAcK, a collection of literature phytochemical data [23], contains 23,127 compounds and 6,544 unique molecular formulae. If a future version of KNApSAcK included all naturally occurring phytochemical compounds (approximately 200,000–400,000 compounds) [25], [26], it is estimated that the number of unique molecular formulae would be 25,000–36,000, based on an extrapolation of the simulated growth curve of KNApSAcK (Fig. 2). Accordingly, the completeness of the current version is estimated to be 19–28% (6,914/36,000 to 6,914/25,000). These results suggest that PubChem is too large and KNApSAcK and KEGG are too small to perform an accurate molecular formula search of plant metabolome data. To further enrich the small databases, additional phytochemical-like formulae were generated in this study through *in silico* “derivatization” of KNApSAcK data, as implemented in LipidBank [27]. Within the large elemental composition space theoretically available, we assumed that a molecular formula located near the current KNApSAcK region should be included in the completed KNApSAcK. For example, hydroxylated (+O) derivatives of each current KNApSAcK entry were likely to be included in the complete KNApSAcK. Many phytochemical-like molecular formulae were generated by hydroxylation (+O), dehydroxylation (−O), methoxylation (+CH_2_O), glucosylation (+C_6_H_10_O_5_), and dehydration (−H_2_O) of existing KNApSAcK entries. In addition, generated elemental compositions not included in the PubChem database were discarded to remove elemental compositions not likely to correspond to actual compounds. Consequently, a new database (called “KNApSAcK plus”) containing a total of 18,312 formulae derived from original and derivatized KNApSAcK entries was created.

### Theoretical background of molecular formula searches

**Figure 3:**
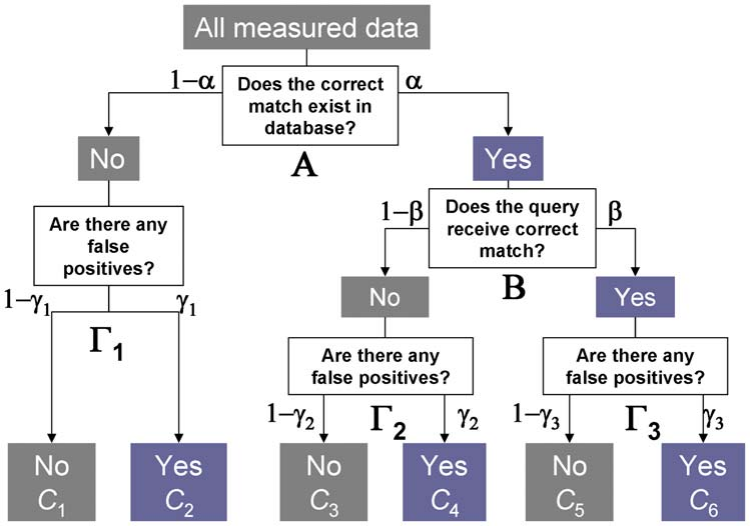
Schematic classification of molecular formula search results. Among a set of queried *m*/*z* values, some will have no matches in a database due to its low completeness (branch point Α, left). For these cases, the results of the molecular formula search should be “no hit” (Class 1, *C*_1_), but false positives will occur in some cases (*C*_2_) (branch point Γ1). Even when the database contains a match (branch point Α, right), some of the queries will fail to receive a correct response (false negatives) due to large analytical errors (branch point B, left). For false negatives, there are two further possibilities (branch point Γ2), including no hits (*C*_3_) and false positives (*C*_4_). Among the queries that receive a correct response (branch point B, right), the most favorable result would be an exact hit without false positives (*C*_5_, branch point Γ3, left). However, additional false positives are likely, depending on the density of the database (*C*_6_, branch point Γ3, right).

Based on the above, the results of a molecular formula search can be divided into six classes [*C*_n_ represents the percentage of queries classified into Class *n* (*n* = 1–6)] by the Yes-No scheme shown in Fig. 3. Here, α, β, γ1, γ2, and γ3 are defined as the branching ratios at Α, Β, Γ1, Γ2, and Γ3 respectively. Among a set of queried *m*/*z* values, some will not have a corresponding entry in the database, due to its low completeness (branch point Α, left). For these cases, the results of the molecular formula search should be “no hit” (Class 1, *C*_1_), but false positives will occur in some cases (*C*_2_) (branch point Γ1). Even when the database contains the correct answers (branch point Α, right), some of the queries will fail to be matched with the correct entries (false negatives) due to large analytical errors (branch point Β, left). For these false negatives, there are two remaining possibilities for the search results (branch point Γ2), including no hits (*C*_3_) and false positives (*C*_4_). Among the queries that receive correct answers (branch point Β, right), the most favorable result is an exact match without false positives (*C*_5_, branch point Γ3, left). However, additional false positives are likely, depending on the density of the database (*C*_6_, branch point Γ3, right).

The percentage of queries that match any molecular formula (total hits, *T*) is defined as: 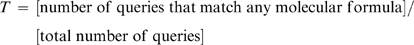
(1)


Theoretically, this percentage can be expressed as:
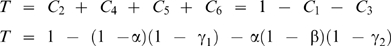
(2)


The percentage of queries that matches only the correct molecular formula (unique hits, *U*) is:
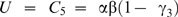



*FDR*, in terms of unique hits, can be defined as follows:
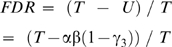
(3)


These equations indicate that *FDR* can be calculated from T and the branching-ratio parameters. In this study, the branching-ratio parameters were determined by employing a Monte Carlo simulation of an elemental composition search of metabolome data, as follows:

- Random sampling was conducted of molecular formulae from the KNApSAcK database.
- Phytochemical-like molecular formulae were generated through random selection of derivatization methods, including hydroxylation (+O), dehydroxylation (−O), methoxylation (+CH_2_O), glucosylation (+C_6_H_10_O_5_), and dehydration (−H_2_O).
- A mass analysis simulation was conducted using the following model:
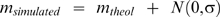
where *m_simulated_* and *m_theol_* represent the simulated and theoretical *m/z* values of protonated molecules, respectively. N(0, σ) is the experimental error of the mass analysis randomly generated using a normal distribution. For the simulation, two assumptions were made: first, that all metabolite signals were molecular weight-related ions with protonated ([M+H]^+^) or deprotonated ([M-H]^−^) forms; and second, that σ was a constant value (mDa). The appropriateness of these assumptions is discussed in the section below.
- The simulated *m*/*z* value was queried as a molecular formula search and the results were classified into the six groups (*C*_1_–*C*_6_) following the scheme shown in Fig. 3.
- Steps (i) to (iv) were repeated in order 100,000 times.
- Branching-ratio parameters were determined.

The branching-ratio parameters were obtained for each molecular formula database for various experimental errors of mass analysis (σ) and searching thresholds (Δ_thres_) listed in Table S1. Among the five branching-ratio parameters (α, β, γ1, γ2,and γ3), it was determined that α was sensitive to the nature of the queried *m*/*z* data (data not shown), indicating that α must be determined for every metabolome data point. Eqn. (2) can be rearranged as follows:
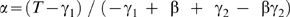
(4)


Thus, α can be determined from the simulated values for β, γ1, and γ2, as well as the value of T obtained using Eqn. (1). Eqn. (3) then becomes:
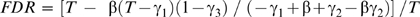
(5)


Thus, *FDR* can be determined from T and the corresponding β, γ1, γ2, and γ3 values previously calculated for each database using the Monte-Carlo simulation (Table S1).

### Determination of FDR for plant metabolome data

Based on the method described above, FDRs for actual plant metabolome data were determined as follows. In a capillary electrophoresis (CE)-TOF/MS dataset of rice seed extracts (*Oryza sativa* L. cv. Sasanishiki), 178 peaks with high-resolution *m*/*z* data were included. The accuracy of mass analysis was deduced to be σ = ∼3 mDa (data not shown); thus, the *m*/*z* data were searched against the KNApSAcK dataset employing a suitable threshold (Δ_thres_ = 2σ, 6 mDa). Among the results, at least one molecular formula was assigned to 98 of the queries. For a KNApSAcK search with mass accuracy σ = 3 mDa and search threshold Δ_thres_ = 6 mDa, the branching-ratio parameters (Table S1) were β = 0.954, γ1 = 0.167, γ2 = 0.249, and γ3 = 0.152. Consequently, *FDR* was determined to be 0.29 using Eqn. (5). The results are shown in Table 1.

**Table 1:** Estimated FDRs for Molecular Formula Search Results.

Dataset	Database	Threshold (Δ)	Queries with hits	Percent total hits (T)	Density (γ_3_)	Completeness (α)	*FDR*	*FDR'* = γ_3_/T
CE-TOF-MS	KNApSAcK	2σ	97	0.54	0.15	0.48	0.29	0.28
		1.5σ	91	0.51	0.11	0.51	0.23	0.21
		1σ	80	0.45	0.06	0.58	0.17	0.13
	KEGG	2σ	119	0.67	0.20	0.64	0.27	0.30
	PubChem	2σ	169	0.95	0.98	0.68	0.99	1.04
	KNApSAcK plus	2σ	134	0.75	0.47	0.68	0.55	0.63
DI-FT-MS	KNApSAcK	2σ	110	0.35	0.009	0.36	0.03	0.03
		1.5σ	101	0.33	0.005	0.37	0.02	0.02
		1σ	97	0.31	0.002	0.46	0.01	0.01
	KEGG	2σ	95	0.31	0.02	0.31	0.05	0.05
	PubChem	2σ	266	0.86	0.83	0.72	0.87	0.97
	KNApSAcK plus	2σ	173	0.56	0.04	0.57	0.07	0.08
LC-TOF-MS	KNApSAcK	2σ	106	0.53	0.33	0.35	0.58	0.62
		1.5σ	84	0.42	0.22	0.28	0.54	0.52
		1σ	63	0.32	0.12	0.28	0.48	0.39
	KEGG	2σ	103	0.52	0.39	0.38	0.57	0.76
	PubChem	2σ	198	0.99	0.99	0.90	1.00	1.00
	KNApSAcK plus	2σ	136	0.68	0.69	0.48	0.79	1.02

Density (γ_3_) values were obtained from Table S1. Percentage of total hits (*T*), completeness (α), *FDR*, and *FDR'* were determined using Eqns. (1), Eqns. (4), Eqns. (5), and Eqns. (7). Results of datasets including CE-TOF-MS (Rice seeds: total number of queries: 178, σ = 3 mDa), DI-FT-MS (*A. thaliana* roots: total number of queries: 310, σ = 0.5 mDa), LC-TOF-MS (*A. thaliana* shoots: total number of queries: 200, σ = 5 mDa) are shown.

Using the same procedure for a KNApSAcK search of direct-infusion (DI)-FT/MS data (σ = 0.5 mDa) derived from the root extract of *Arabidopsis thaliana*, *FDR* was estimated to be 0.03. In addition, *FDR* was deduced to be 0.58 for liquid chromatography (LC)-Q-TOF/MS data (σ = 5 mDa) for *A. thaliana* shoot metabolites (Table 1).

These results indicate that the reliability of the KNApSAcK search results for TOF-MS data (σ = 3–5Da) was relatively low (Table 1). Narrower search thresholds (Δ_thres_ = 1 or 1.5 σ) did not result in substantial improvements in annotation quality, as demonstrated by the LC-Q-TOF/MS data (Table 1). Thus, elemental composition search results for TOF-MS data should be carefully applied, considering additional structural information, when interpreting metabolome data. It has also been suggested that one of the most straightforward ways to improve FDR is to improve the accuracy of mass analyses. Mass analysis accuracy at FT-MS levels (σ = ∼0.5–1 mDa) is likely required to obtain a molecular formula search with a low FDR value (<10%; Table 1).

### Method validation

The methodology for estimating FDRs, as described in this study, is based on branching-ratio parameters determined by a Monte Carlo simulation of an elemental composition search. Thus, validation of the two assumptions employed for simplification of the simulation model is required to ensure the accuracy of the estimated FDRs.

The first assumption was that all metabolite signals were considered to be molecular weight-related ions with protonated ([M+H]^+^) or deprotonated ([M–H]^−^) forms. It is generally expected that other types of ions—such as various adducts or fragment ions—are present in the actual metabolome data. The low completeness (α = 0.68) of the PubChem search results for the CE-TOF/MS dataset (Table 1) was probably due to such irregular ions. However, the presence of irregular ions does not affect the estimated FDRs, since molecular formula of irregular ions such as sodium adduct ([M+Na]^+^) are not included in databases and it affected only the completeness parameter (α) in Figure 3. As noted above, the parameter α was determined for every search using Eqn. (4).

**Figure 4:**
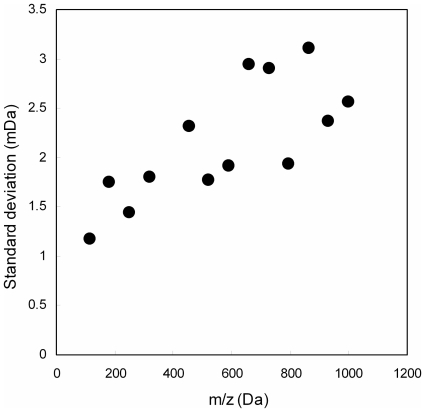
Mass-to-charge ratio (*m/z*) dependency of the accuracy of mass analysis. Standard deviations were calculated from a series of *m*/*z* values for [nHCOONa+H]^+^ ions obtained using the direct infusion mode of LC-Q-TOF-MS (Waters Corp.).

The second assumption was that the mass analysis error (mDa) can be considered constant. Although analytical error in the field of mass spectrometry is commonly expressed as parts per million (ppm) [6], mass accuracy can be significantly affected by mass to charge ratio, concentration of the compound, and the amounts of co-eluting metabolites. Indeed, the measured errors acquired using TOF-MS (Q-Tof Premier, Waters) were not proportional to the *m*/*z* values (Fig. 4). This suggests that the absolute unit (mDa) rather than the relative unit (ppm) is more suitable for defining threshold values for molecular formula searches. However, this second assumption is not valid in the strictest sense. In this study, to evaluate the appropriateness of the FDRs estimated on the basis of this simplified model, search results for the CE-TOF/MS dataset (database: KNApSAcK, σ = 3 Da, Δ_thres_ = 6 Da, Table 1) were compared with annotation information for authentic compounds. The elemental compositions of 59 peaks out of 97 hits were confirmed matches to those of authentic standards (data not shown). Because metabolite annotations by authentic compounds are still incomplete, the *FDR* deduced from the results [(98−59)/98 = 0.39] is roughly consistent with the estimated FDR (*FDR* = 0.29), suggesting that the second assumption is reasonably valid for estimation of FDRs.

### Performance of isotope ratio filtering

Recently, it has been suggested that isotope ratio filtering may be a useful approach for improving FDR [1], [3]. Metabolites derived from living organisms contain naturally occurring stable isotopes. Because the theoretical ratio (*i* = [M+1]/[M]) of single stable isotope-labeled molecules (M+1) to monoisotopic molecules (M) is unique to each formula, the false positive rate of the molecular formula search can be reduced through comparison of theoretical and measured *i* values. Indeed, it has been reported that isotope ratio filtering is key to reducing the number of candidate molecular formulae when searching an artificial database [17]. Moreover, the isotope filtering technique has been employed for annotating actual metabolome data [1], [3]. However, the performance of isotope filtering and the accuracy required for determining the isotope ratio have not been well investigated [1], [17], [28].

To investigate the potential of isotope ratio filtering, the Monte Carlo simulations described above were repeated. Following generation of phytochemical-like molecular formulae from KNApSAcK via random selection of a derivatization method, simulated *m*/*z* values were searched against the KNApSAcK and PubChem databases, employing accuracies of mass analysis and search thresholds corresponding to FT-MS (σ = 1 Da and Δ_thres_ = 2 Da) and TOF-MS (σ = 5 Da and Δ_thres_ = 10 Da). For false positives at the branch points Γ1–Γ3 in the scheme shown in Fig. 3, the ratios (*i* = [M+1]/[M]) of single stable isotope-labeled molecules (M+1) to monoisotopic molecules (M) of the query formula (*i*_Q_) and those of the false positives (*i*_FP_) were estimated using the following equation: 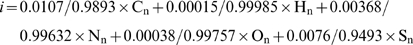
where C_n_, H_n_, N_n_, O_n_, and S_n_ represent the numbers of these atoms in the composite formula.

**Figure 5:**
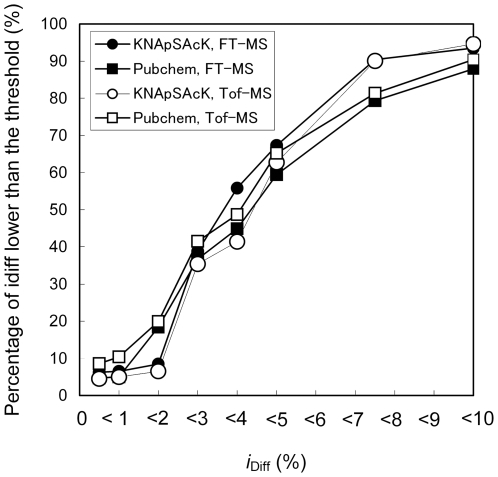
Absolute differences between the theoretical isotope ratios of the query formulae and the false positives. Results are shown for molecular formulae in the KNApSAcK (circles) and PubChem compound (squares) datasets, with FT-MS (σ = 1 ppm and Δ_thres_ = 2 ppm, closed symbols) and TOF-MS (σ = 5 ppm and Δ_thres_ = 10 ppm, open symbols) accuracies of mass analyses. The x-axis represents the *i*_diff_ (*i*_diff_ = |*i*_Q_−*i*_FP_ |) threshold and the y-axis represents the percentage of *i*_diff_ lower than the threshold among 1,000 pairs of query formulae and false positives.

The integrated frequencies of the absolute differences (*i*_diff_ = |*i*_Q_−*i*_FP_ |) between *i*_Q_ and *i*_FP_ are shown in Fig. 5. Half of the *i*_diff_ values (y-axis) for false positives were <∼4% (x-axis) and nearly all of the *i*_diff_ values were <10% (Fig. 5). The trends were independent of both the target database and the search thresholds. Therefore, highly accurate isotope ratio determination (σ<2%) is needed to obtain a 50% reduction in the frequency of false positives at branch points Γ1–Γ3. These results indicate that isotope ratio filtering can be effective in screening candidate molecular formulae when high-quality data with exact isotope ratios are available.

### Properties of elemental composition databases for reliable searching

**Figure 6:**
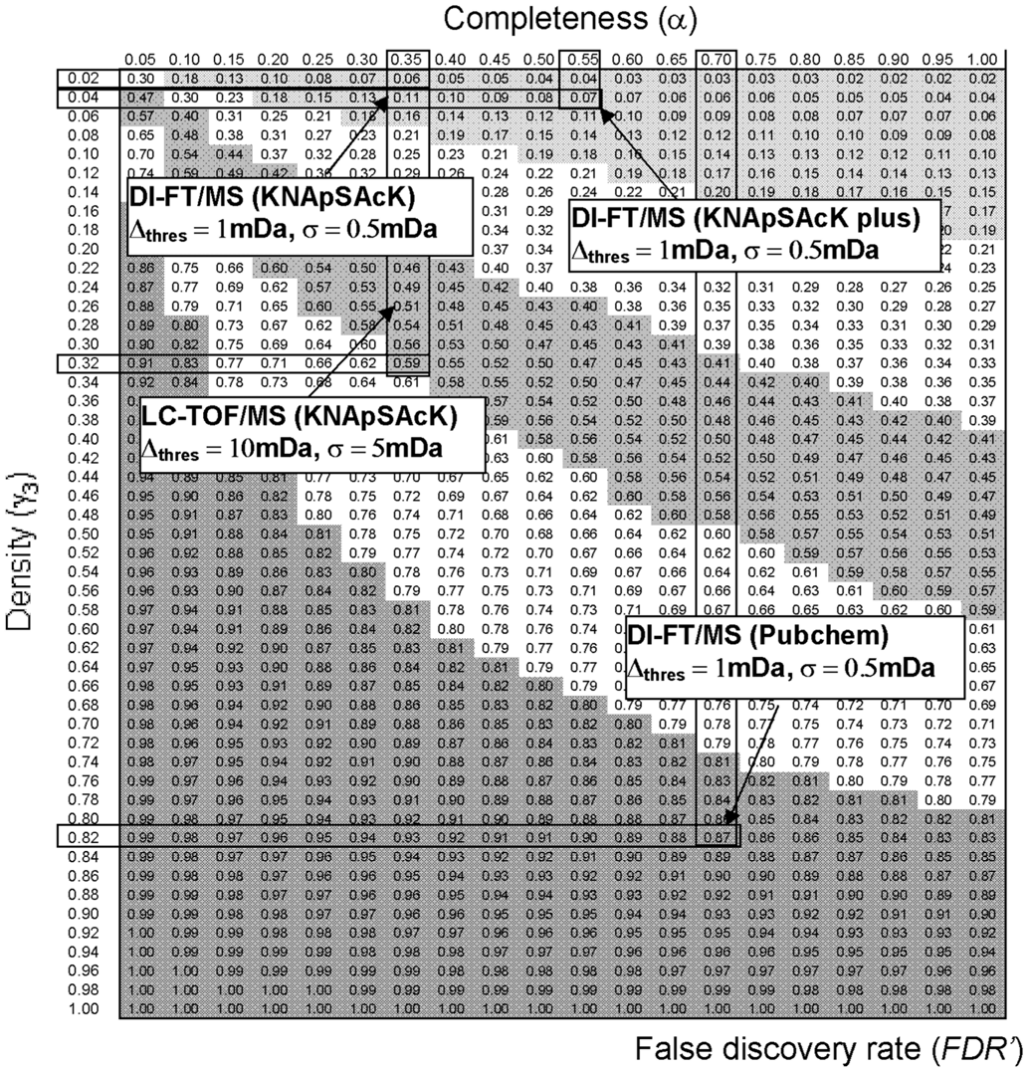
Relationships among the false discovery rate (FDR'), completeness (α), and density (γ_3_). A search threshold Δ_thres_ = 2σ was used. The estimated FDR's can be obtained by cross-checking the corresponding columns (α) and rows (γ_3_). Results for four representative cases are shown.

The two concepts of database, completeness and density, have been introduced in this study to describe the properties of molecular formula databases, corresponding to the parameters α and γ_1–3_ in the classification scheme shown in Fig. 3, respectively. When γ_3_ = γ_1_ = γ_2_ is assumed, Eqn. (5) can be simplified as follows:
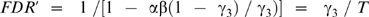
(7)where *FDR'* represents an estimated FDR obtained using the simplifying assumption described above. Eqn. (7) indicates that *FDR'* can be calculated from the two parameters α and γ3, as β is always 0.95 when Δ_thres_ is set to 2σ. The relationships among *FDR'*, completeness (α), and density (γ3) in the elemental composition search results are shown in Fig. 6. For example, for α and γ3 values for a KNApSAcK search of LC-TOF/MS data (Δ_thres_ = 10 mDa, σ = 5 mDa) of 0.35 and 0.33, respectively (Table 1), *FDR'* can be determined to be 0.59 by looking up the corresponding column (α = 0.35) and row (γ3 = 0.32) as shown in Fig. 6. The *FDR'* value obtained (0.59) is essentially the same as that of *FDR* (0.58) determined by the original procedure.

Fig. 6 indicates that *FDR'* could be improved by lower density (γ3) and higher completeness (α) in the database. The former can clearly be attained by searching mass spectrometry data with a higher accuracy of mass analysis such as FT-MS. A fairly good *FDR* (0.03) was obtained for the KNApSAcK search of the DI-FT-MS dataset (α = 0.36, γ3 = 0.009; Table 1 and Fig. 6), primarily due to the low γ3 values achieved by employing a narrow threshold value for searching (Δ_thres_ = 1 mDa, σ = 0.5 mDa). The latter strategy (higher completeness, α) requires further enrichment of the molecular formula database, which is inevitably accompanied by an increase in the density of the database (γ3). Indeed, molecular formula searches of the DI-FT/MS dataset using PubChem afforded results with high completeness (α = 0.72). However, the FDRs were at unacceptable levels for practical use (0.87) because of the very high γ3 values (0.83) associated with the high density of the database (Table 1 and Fig. 6). Thus, the quality of the molecular formula search results depends greatly on the properties of the database. It is also apparent that a database with high completeness (α) and low density (γ3) would be preferable for performing a high-quality search. Therefore, molecular formula databases should not include useless entries, and a small, customized compound database suitable for the specific research purpose is preferable to a large, all-in-one database.

However, the completeness of the current KNApSAcK and KEGG Compound databases are not sufficient for fully annotating metabolome data. Thus, derivatization of the molecular formula database was attempted by creating KNApSAcK plus. The results of the molecular formula search indicated that completeness (α) had improved compared to the original KNApSAcK (Table 1 and Fig. 6). However, FDR also significantly increased, suggesting that the number of useless entries was increased by database derivatization.

## Discussion

A novel method for evaluating the FDR of the results of an elemental composition search of metabolome data obtained by MS is presented in this paper. Based on the FDR analyses, several aspects of an elemental composition search, including setting a threshold, estimating FDR, and the types of elemental composition databases most reliable for searching are discussed in the following sections.

### Setting a search threshold

To maintain a false negative rate of 5%, the threshold for searching (Δ_thres_) must be twice the standard deviation of the analytical error of mass analysis (σ). Thus, evaluation of the mass analysis accuracy of the queried metabolome data is essential before performing an elemental composition search. Applications of lower thresholds for searching (Δ_thres_ = 1 or 1.5 σ) did not substantially improve low-quality annotations, as demonstrated for the LC-Q-TOF/MS data (Table 1).

### Estimating FDR

In this study, we developed a novel method for determining FDRs of molecular formula search results that can be applied to actual metabolome data (Table 1). The methodology is based on branching-ratio parameters determined through a Monte Carlo simulation of elemental composition searches. Although the simulation model employed in this study has been validated, it is expected that the estimated FDRs contain some error derived from the simplifying assumptions of the simulation model. Further improvements in the simulation model of mass analysis will enable more exact estimation of FDRs.

The evaluation of FDRs in plant metabolome data indicated that, although accurate mass data obtained by TOF-MS have been widely used in assigning elemental compositions, a careful treatment of search results is required to preclude incorrect interpretations of metabolome data because of the relatively high FDRs for these results [9]. On the other hand, annotations of FT-MS data are sufficiently reliable for searches of relatively small databases. Recently, Orbitrap mass spectrometers have been employed for metabolome analyses [2], [29]–[31]. Although metabolome data derived from Orbitrap-MS were not analyzed in this study, the high mass accuracy (1–5 ppm) of the analyzer should allow a molecular formula search with low FDR. FDRs for elemental composition search results of Orbitrap data can be estimated by the present method because the branching-ratio parameters for high mass accuracy data are available in Table S1.

### Features of elemental composition databases supporting reliable searching

The estimated FDRs indicated that the quality of search results can be improved not only by performing more accurate mass analysis but also by modifying the properties of the compound database (Table 1). The low percentages of total hits (T) for the KEGG and KNApSAcK searches were a result of the low completeness of these databases, and it has been theoretically determined that FDR levels can be improved by using databases with higher completeness (Fig. 6). This requires further enrichment of the molecular formula database entries. However, such enrichment is inevitably accompanied by an increase in the density of the database, resulting in higher FDRs (Table 1, Fig. 6). Thus, the molecular formula database should not include useless entries. For example, synthetic drug entries in PubChem would reduce the effectiveness of a plant metabolome data search. Conversely, phytochemical entries would be useless for studies analyzing residual drugs in food samples, suggesting that a small, custom-made compound library suitable for the specific research purpose is preferable to a large, all-in-one database. However, as noted above, compound entries in customized databases such as KNApSAcK are currently not sufficient for full annotation of metabolome data (Table 1). This problem was overcome, at least in part, by using KNApSAcK plus, which was generated using *in silico* derivatization of the metabolite database, as implemented in LipidBank [27]. The levels of T improved from the original KNApSAcK, but the FDRs also slightly increased (Table 1). These results indicate that one of the most useful methods for developing a high-quality database for plant metabolomics study is careful collection of phytochemical as well as species-metabolite relationship information, such as in KNApSAcK [23].

It is noteworthy that elemental composition search is not sufficient for full elucidation of metabolite structure. Additional information, such as isotope filtering [1], [3], determination of the carbon number using plant samples grown on ^13^C-labeled medium [32], [33], and application of tandem mass spectra data [34]–[37], facilitate determination of a unique molecular formula. Among these methods, this study demonstrated that exact determination of an isotope ratio is required to perform effective isotope filtering (Fig. 5). Even if a single formula is deduced, additional tandem mass spectra data as well as literature information is required for more detailed metabolite elucidation from among the many possible structural isomers [38]–[41]. However, the elemental composition search is important as the first step of metabolite annotation. An assessment of FDR and improving the quality of elemental composition search results is one basis for characterizing, annotating, and further identifying metabolite signals in metabolome data.

## Materials and Methods

### Development of compound databases

The PubChem Compound (08/07/15 version) and KEGG Compound (08/08/15 version) datasets were obtained from the NIH (http://pubchem.ncbi.nlm.nih.gov/) and KEGG (http://www.genome.jp/kegg/) web sites, respectively. The KNApSAcK (KS, 08/08/22 version) dataset was produced by our group and is available online (http://kanaya.aist-nara.ac.jp/KNApSAcK/). Following the removal of non-small molecule entries, structural isomers with identical molecular formulae were combined into one entry. To remove manmade compounds, lists of molecular formula comprising C, H, N, O, S, and P were obtained and used for the analyses. All data processing was performed using in-house Perl scripts and Microsoft Excel 2002.

### Determination of FDRs

The FDRs for the elemental composition search results of actual metabolome data were determined using the following procedures:

- Evaluate the mass analysis accuracy of the metabolome data (σ).
- Perform a molecular formula search against each of the databases using a suitable threshold value (Δ_thres_ = σ, 1.5σ, or 2σ).
- Calculate the parameter *T* using Eqn. (1).
- Determine *FDR* using Eqn. (3) and the values for the branching-ratio parameters (β, γ1, γ2, and γ3) listed in Table S1.

### Metabolome analyses

Dehulled rice seeds (*Oryza sativa* cv. Sasanishiki) were extracted and analyzed using CE-TOF/MS as previously described [42]. DI-FT/MS data were acquired as part of a previous study [12]. *Arabidopsis thaliana* (Col-0 ecotype) seedlings were grown on 1/2 MS medium plates at 20°C with a 16-h daily photoperiod. Two weeks after germination, whole tissues of 20 seedlings were collected, weighed, and used for the metabolic profiling analysis of LC-Q-TOF/MS (Q-Tof Premier, Waters Corp. Milford, MA), as previously described [9].

## Supporting Information

Table S1 Branching ratio parameters of A: KEGG Compound, B: KNApSAcK, C: PubChem Compound, and D: KNApSAcK plus at various accuracies of mass analysis and thresholds for searching.
(0.18 MB DOC)
