# Supplementary material for: Assessment of Metabolome Annotation Quality: A Method for Evaluating the False Discovery Rate of Elemental Composition Searches
Source: PLoS One. 2009 Oct 16;4(10):e7490. doi: 10.1371/journal.pone.0007490 (PMC2761541; doi:10.1371/journal.pone.0007490)
Supplement: Table S1 — Branching ratio parameters of A: KEGG Compound, B: KNApSAcK, C: PubChem Compound, and D: KNApSAcK plus at various accuracies of mass analysis and thresholds for searching. (0.18 MB DOC) [file pone.0007490.s001.doc]

Table S1

| A: KEGG Compound | | | | | |
| --- | --- | --- | --- | --- | --- |
| Accuracy (σ, mDa) | Threshold Δ_thres_  mDa) | β | γ_1_ | γ_2_ | γ_3_ |
| 0.1 | 0.2 | 0.955 | 0.001 | 0.000 | 0.001 |
| 0.5 | 0.25 | 0.383 | 0.002 | 0.005 | 0.001 |
| 0.5 | 0.5 | 0.684 | 0.006 | 0.012 | 0.005 |
| 0.5 | 0.75 | 0.865 | 0.009 | 0.024 | 0.012 |
| 0.5 | 1 | 0.956 | 0.014 | 0.026 | 0.017 |
| 1 | 0.5 | 0.383 | 0.008 | 0.014 | 0.004 |
| 1 | 1 | 0.688 | 0.016 | 0.025 | 0.019 |
| 1 | 1.5 | 0.864 | 0.026 | 0.053 | 0.035 |
| 1 | 2 | 0.952 | 0.038 | 0.085 | 0.047 |
| 2 | 4 | 0.955 | 0.088 | 0.154 | 0.131 |
| 3 | 1.5 | 0.379 | 0.035 | 0.060 | 0.034 |
| 3 | 3 | 0.689 | 0.069 | 0.129 | 0.094 |
| 3 | 4.5 | 0.864 | 0.104 | 0.189 | 0.149 |
| 3 | 6 | 0.958 | 0.140 | 0.312 | 0.204 |
| 4 | 8 | 0.954 | 0.187 | 0.444 | 0.299 |
| 5 | 2.5 | 0.390 | 0.065 | 0.118 | 0.073 |
| 5 | 5 | 0.683 | 0.125 | 0.271 | 0.167 |
| 5 | 7.5 | 0.864 | 0.181 | 0.432 | 0.278 |
| 5 | 10 | 0.955 | 0.235 | 0.518 | 0.390 |
| 10 | 20 | 0.955 | 0.397 | 0.770 | 0.684 |

| B: KNApSAcK | | | | | |
| --- | --- | --- | --- | --- | --- |
| Accuracy (σ, mDa) | Threshold Δ_thres_  mDa) | β | γ_1_ | γ_2_ | γ_3_ |
| 0.1 | 0.2 | 0.951 | 0.001 | 0.002 | 0.000 |
| 0.5 | 0.25 | 0.381 | 0.002 | 0.002 | 0.001 |
| 0.5 | 0.5 | 0.683 | 0.005 | 0.006 | 0.002 |
| 0.5 | 0.75 | 0.865 | 0.008 | 0.010 | 0.005 |
| 0.5 | 1 | 0.952 | 0.013 | 0.010 | 0.009 |
| 1 | 0.5 | 0.377 | 0.008 | 0.008 | 0.002 |
| 1 | 1 | 0.684 | 0.017 | 0.016 | 0.010 |
| 1 | 1.5 | 0.867 | 0.025 | 0.028 | 0.018 |
| 1 | 2 | 0.953 | 0.037 | 0.052 | 0.026 |
| 2 | 4 | 0.954 | 0.105 | 0.118 | 0.088 |
| 3 | 1.5 | 0.376 | 0.040 | 0.045 | 0.019 |
| 3 | 3 | 0.680 | 0.082 | 0.105 | 0.058 |
| 3 | 4.5 | 0.864 | 0.125 | 0.152 | 0.108 |
| 3 | 6 | 0.954 | 0.167 | 0.249 | 0.152 |
| 4 | 8 | 0.955 | 0.228 | 0.421 | 0.235 |
| 5 | 2.5 | 0.378 | 0.074 | 0.094 | 0.045 |
| 5 | 5 | 0.681 | 0.152 | 0.221 | 0.120 |
| 5 | 7.5 | 0.865 | 0.226 | 0.396 | 0.217 |
| 5 | 10 | 0.955 | 0.290 | 0.507 | 0.330 |
| 10 | 20 | 0.955 | 0.484 | 0.754 | 0.655 |

| C: PubChem compound | | | | | |
| --- | --- | --- | --- | --- | --- |
| Accuracy (σ, mDa) | Threshold Δ_thres_  mDa) | β | γ_1_ | γ_2_ | γ_3_ |
| 0.1 | 0.2 | 0.955 | 0.149 | 0.251 | 0.253 |
| 0.5 | 0.25 | 0.383 | 0.225 | 0.470 | 0.326 |
| 0.5 | 0.5 | 0.681 | 0.091 | 0.702 | 0.598 |
| 0.5 | 0.75 | 0.865 | 0.444 | 0.863 | 0.743 |
| 0.5 | 1 | 0.954 | 0.509 | 0.887 | 0.832 |
| 1 | 0.5 | 0.382 | 0.374 | 0.757 | 0.603 |
| 1 | 1 | 0.684 | 0.521 | 0.896 | 0.845 |
| 1 | 1.5 | 0.865 | 0.608 | 0.940 | 0.919 |
| 1 | 2 | 0.955 | 0.663 | 0.958 | 0.950 |
| 2 | 4 | 0.953 | 0.784 | 0.979 | 0.982 |
| 3 | 1.5 | 0.380 | 0.624 | 0.939 | 0.917 |
| 3 | 3 | 0.682 | 0.741 | 0.972 | 0.972 |
| 3 | 4.5 | 0.866 | 0.807 | 0.982 | 0.983 |
| 3 | 6 | 0.953 | 0.845 | 0.992 | 0.989 |
| 4 | 8 | 0.954 | 0.869 | 0.995 | 0.992 |
| 5 | 2.5 | 0.381 | 0.717 | 0.966 | 0.964 |
| 5 | 5 | 0.681 | 0.821 | 0.988 | 0.985 |
| 5 | 7.5 | 0.864 | 0.867 | 0.994 | 0.991 |
| 5 | 10 | 0.955 | 0.898 | 0.995 | 0.995 |
| 10 | 20 | 0.955 | 0.948 | 0.998 | 0.998 |

| D: KNApSAcK plus | | | | | |
| --- | --- | --- | --- | --- | --- |
| Accuracy (σ, mDa) | Threshold Δ_thres_  mDa) | β | γ_1_ | γ_2_ | γ_3_ |
| 0.1 | 0.2 | 0.952 | 0.003 | 0.001 | 0.001 |
| 0.5 | 0.25 | 0.380 | 0.010 | 0.009 | 0.002 |
| 0.5 | 0.5 | 0.680 | 0.005 | 0.026 | 0.010 |
| 0.5 | 0.75 | 0.867 | 0.032 | 0.056 | 0.024 |
| 0.5 | 1 | 0.954 | 0.030 | 0.061 | 0.043 |
| 1 | 0.5 | 0.383 | 0.032 | 0.036 | 0.011 |
| 1 | 1 | 0.682 | 0.061 | 0.071 | 0.050 |
| 1 | 1.5 | 0.866 | 0.096 | 0.120 | 0.082 |
| 1 | 2 | 0.953 | 0.087 | 0.204 | 0.116 |
| 2 | 4 | 0.954 | 0.196 | 0.410 | 0.320 |
| 3 | 1.5 | 0.381 | 0.129 | 0.170 | 0.082 |
| 3 | 3 | 0.680 | 0.239 | 0.338 | 0.223 |
| 3 | 4.5 | 0.864 | 0.336 | 0.440 | 0.364 |
| 3 | 6 | 0.952 | 0.271 | 0.604 | 0.474 |
| 4 | 8 | 0.952 | 0.350 | 0.709 | 0.594 |
| 5 | 2.5 | 0.382 | 0.223 | 0.300 | 0.181 |
| 5 | 5 | 0.681 | 0.384 | 0.519 | 0.404 |
| 5 | 7.5 | 0.867 | 0.505 | 0.681 | 0.558 |
| 5 | 10 | 0.954 | 0.399 | 0.784 | 0.691 |
| 10 | 20 | 0.954 | 0.549 | 0.896 | 0.873 |
